# Mulberry Polyphenols Ameliorate Metabolic Abnormalities Accompanied by Spatial Microbial and Short-Chain Fatty Acid Alterations in Type 2 Diabetic Mice

**DOI:** 10.3390/foods15193568

**Published:** 2026-10-08

**Authors:** Tong Zhang, Ping-Ping Wang, Abdulla Yusuf, Fu Xiong, Chun Chen

**Affiliations:** 1 The Key Laboratory of Xinjiang Native Medicinal and Edible Plant Resources Chemistry, College of Chemistry and Environmental Science, Kashi University, Kashi 844000, China; 2 School of Food Science and Engineering, South China University of Technology, Guangzhou 510640, China; lfxfu@scut.edu.cn; 3 School of Chemical Engineering and Light Industry, Guangdong University of Technology, Guangzhou 510006, China; 4 South China University of Technology-Zhuhai Institute of Modern Industrial Innovation, Zhuhai 519175, China; 5 Overseas Expertise Introduction Center for Discipline Innovation of Food Nutrition and Human Health (111 Center), Guangzhou 510640, China

**Keywords:** mulberry polyphenols, gut microbiota, type 2 diabetes mellitus, short-chain fatty acids, spatiotemporal heterogeneity

## Abstract

The gut microbiota may contribute to the anti-diabetic effects of mulberry polyphenols (MPs), although the associated spatial microbial patterns and metabolic alterations remain unclear. We investigated microbiota composition in fecal, cecal, and colonic samples and temporal changes in fecal short-chain fatty acids (SCFAs) during an 8-week MP intervention in type 2 diabetic (T2DM) mice using 16S rRNA sequencing and gas chromatography. MPs effectively reduced fasting blood glucose and improved diabetes-associated pathological alterations. Spatially, MP administration was associated with distinct changes in the ascending colonic microecology, characterized by an increased relative abundance of *Allobaculum* in the lumen and a 23.17% reduction in the relative abundance of mucosa-associated *Helicobacter*. At the phylum level, MPs increased fecal Bacteroidetes by 7.16% and reduced the relative abundance of Actinobacteria in the cecal, transverse, and descending colonic contents (decreased by 17.03%, 27.24%, and 15.08%, respectively). Temporally, MP administration was associated with time-dependent changes in fecal SCFA concentrations, especially acetate, butyrate, and isobutyrate. Collectively, MP administration was associated with improved glycemic control and coordinated spatial microbial alterations and temporal SCFA changes, characterized by pronounced microbial compositional changes in the ascending-colon region and time-dependent increases in SCFA concentrations over time. These findings provide insights into polyphenol-associated microbiota alterations and support further investigation of the microbial and metabolic responses to MPs in T2DM.

## 1. Introduction

Type 2 diabetes mellitus (T2DM) is a complex metabolic disorder characterized by insulin resistance and disrupted glucose and lipid homeostasis [1]. It is frequently accompanied by severe complications such as cardiovascular, kidney, and liver disease [2], which substantially increase patient mortality and the global public health burden. Although conventional hypoglycemic agents effectively control blood glucose, their long-term use is often limited by adverse effects, particularly gastrointestinal disturbances [3]. In recent years, gut microbiota dysbiosis has emerged as a critical mediator in T2DM pathogenesis. An imbalance in gut microbial composition and their metabolites, such as short-chain fatty acids (SCFAs), can compromise the intestinal barrier and trigger systemic low-grade inflammation, thereby exacerbating metabolic disorders [4,5]. Consequently, remodeling the gut microbiota and restoring metabolic homeostasis have become frontier targets for T2DM prevention and therapy [6,7].

Dietary polyphenols have shown considerable potential in improving glycolipid metabolism and mitigating T2DM, owing to their natural advantages including multi-target regulation, high biosafety, and minimal systemic adverse effects [8]. However, their limited systemic bioavailability (typically <10%) can result in substantial intestinal exposure to intact polyphenols [9], allowing gut microbiota-mediated biotransformation to contribute to, rather than exclusively determine, their biological effects [10]. This metabolic characteristic also applies to mulberry polyphenols (MPs), which possess well-documented hypoglycemic properties [11]. MPs exert prebiotic effects that modulate gut microbiota composition [12], while one of the representative bioactive components of mulberry polyphenols, cyanidin-3-O-glucoside (C3G) [13], can undergo both host absorption and metabolism and microbial biotransformation into bioactive metabolites [14]. Importantly, polyphenols may also act directly within the gastrointestinal tract by modulating intestinal epithelial and enteroendocrine functions, digestive enzymes, glucose transport, and inflammatory and redox-sensitive pathways [15,16]. In addition, adaptive stress responses involving Nrf2- and AMPK-related signaling may represent complementary host mechanisms, consistent with a potential hormetic mode of action [17,18]. Thus, both host-directed and microbiota-associated pathways may contribute to the anti-diabetic effects of MPs.

In particular, the interaction between polyphenols and gut microbiota has been extensively investigated [19]; current mechanistic studies predominantly rely on endpoint fecal samples, which suffer from substantial limitations in spatiotemporal resolution. Spatially, conventional fecal sequencing merely reflects the terminal admixture of the digestive tract, obscuring the core microecological niches from the stomach to various colonic segments and the mucus layer that are responsible for polyphenol biotransformation [16,20]. Multi-compartment profiling can provide complementary information on spatial microbiota–metabolite associations beyond fecal measurements alone [21]. Temporally, single-time-point static observations fail to capture the dynamic successional trajectory from the initial metabolite accumulation to eventual homeostatic reconstruction [22]. Given the established anti-diabetic efficacy and intestinal enrichment characteristics of MPs, we hypothesize that their microecological modulatory effects in ameliorating T2DM exhibit pronounced spatiotemporal heterogeneity.

Thus, we dynamically evaluated distinct cecal and colonic segments and mucosal layers across different intervention stages in a T2DM mouse model. This study aims to characterize spatial patterns of MP-associated changes in gut microbiota and temporal alterations of SCFAs in T2DM mice and to explore their potential relationships with metabolic improvement. Our findings provide a high-resolution description of the spatiotemporal microbial and SCFA responses to MPs and generate hypotheses for future studies investigating the mechanisms underlying their metabolic effects.

## 2. Materials and Methods

### 2.1. Material and Chemicals

Mulberry was provided by Urumqi Silk Fruit Road Trading Co., Ltd. (Urumqi, China). Inulin was obtained from Shanghai Yuanye Bio-Technology Co., Ltd. (Shanghai, China). A sixty-percent high-fat model diet was purchased from Nantong Trophy Feed Technology Co., Ltd. (Nantong, China). Streptozotocin was obtained from Sigma-Aldrich (St. Louis, MO, USA). Quant-iT PicoGreen dsDNA Assay Kit was purchased from Invitrogen (Carlsbad, CA, USA). DNA extraction kit was obtained from Omega Bio-tek, Inc. (Norcross, GA, USA). All other reagents used were of analytical grade.

### 2.2. Extraction of Mulberry Polyphenols (MPs)

**Figure 1:**
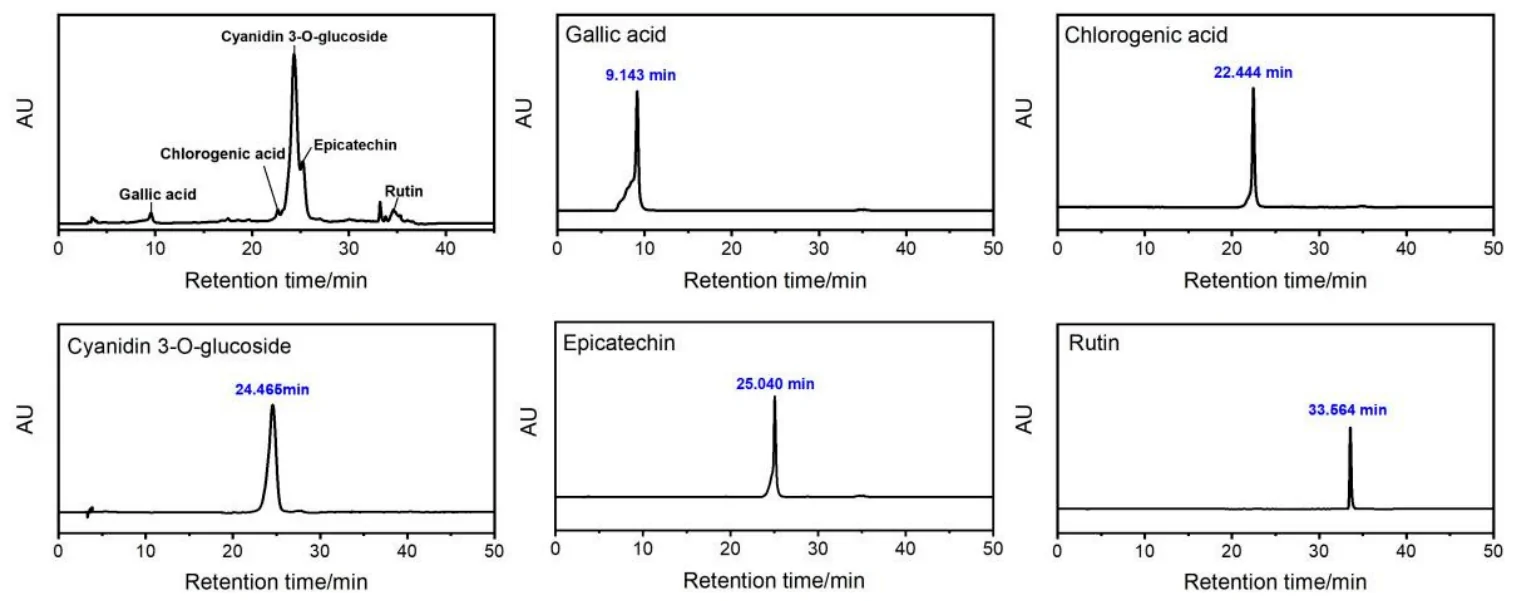
HPLC chromatographic identification of MPs and reference standards (gallic acid, chlorogenic acid, cyanidin-3-O-glucoside, epicatechin, and rutin).

Mulberries were homogenized in pre-cooled 75% acetone with 1% HCl (1:10, *w*/*v*) for 5 min, centrifuged (3000 rpm, 5 min), and concentrated at 45 °C. The extract was precipitated with three volumes of ethanol at 4 °C for 24 h, and further centrifuged (10,000 rpm, 20 min), filtered through a 0.45 μm filter membrane, and freeze-dried using a freeze dryer (18-N, Ningbo Scientz Biotechnology Co., Ltd., Ningbo, China) to obtain the crude extract. The crude extract was redissolved in acidified water (pH 5.0, 1:20, *w*/*v*) and mixed with Amberlite XAD-2 macroporous resin (4:1, *v*/*w*) under shaking conditions (150 rpm, 30 °C) for 12 h to allow polyphenol adsorption. The resin was subsequently desorbed with 50% ethanol for 12 h. The desorbed fraction was freeze-dried and stored at −20 °C for animal administration. The chemical characteristics of the obtained MP preparation were further evaluated. The total polyphenol content and total flavonoid content were 37.42 ± 0.44 mg gallic acid equivalents/g and 18.37 ± 2.70 mg rutin equivalents/g, respectively. HPLC analysis confirmed the presence of representative phenolic compounds, including gallic acid, chlorogenic acid, cyanidin-3-O-glucoside, epicatechin, and rutin (Figure 1).

### 2.3. Animal Experiments

Male C57BL/6J mice (weighing 18 ± 2 g, 7 weeks old) were housed in the SPF-grade laboratory of the Animal Center of Guangdong Pharmaceutical University. All animal experiments were conducted in accordance with the Guide for the Care and Use of Laboratory Animals and were approved by the Animal Ethics Committee of Guangdong Pharmaceutical University (Approval No. gdpulacspf 2017505, date of approval: 22 March 2022). Male C57BL/6J mice were housed at four animals per cage with poplar wood shavings used as bedding material. Cage positions on the rack were randomly assigned among experimental groups to minimize potential positional effects. The mice were housed under controlled environmental conditions (temperature 23 ± 1 °C, humidity 55 ± 5%) with a 12 h light/dark cycle. The animals had free access to food and sterile drinking water throughout the experiment. No specific humane endpoints were predefined for this study. Animals were routinely monitored for their general health status throughout the experiment, with assessments performed at least once every 3 days. After one week of acclimatization, the mice were fed a high-fat diet (HFD) for 4 weeks. T2DM was induced by intraperitoneal injection of streptozotocin (STZ) at a dose of 50 mg/kg after HFD. The HFD/STZ-induced T2DM model was selected because it combines diet-induced metabolic stress with partial β-cell dysfunction and is widely used for investigating T2DM-associated metabolic alterations and nutritional interventions [23]. After 7 days of STZ injection, the mice were fasted for 12 h, and FBG levels were measured using an Accu-Chek Performa glucose meter (ACCU-CHEK Performa, Mannheim, Germany). FBG levels above 11.1 mM were considered indicative of successful T2DM model establishment. All animals met the predefined criteria for successful T2DM model establishment, and no animals or data points were excluded from the subsequent analyses.

The modeled T2DM animals were randomly allocated using a random number table into three groups (*n* = 10 per group), including the model group, MP group (500 mg/kg bw), and inulin group (5 g/kg bw). MPs were dissolved in sterile saline at a concentration of 40 mg/mL and the gavage volume was adjusted individually according to body weight measured before dosing, corresponding to 12.5 mL/kg bw. The dose of MPs was selected with reference to previous preclinical studies using comparable doses of mulberry polyphenols in diabetic mouse models, in which 400 mg/kg bw produced significant metabolic and gut microbiota-modulatory effects [12]. The dose of inulin was selected based on previous studies using 50 mg/kg bw in HFD/STZ-induced diabetic mice [24]. A normal control group (*n* = 10) was also established. No blinding was applied during the allocation, experimental procedures, outcome assessment, or data analysis. The sample size was determined with reference to previously published studies using comparable T2DM mouse models. Each individual mouse was considered the experimental unit. The MPs and inulin groups were administered their respective treatments via oral gavage once daily, while the control and model groups received an equal volume of sterile saline once daily. This intervention continued for 8 consecutive weeks. The body weight, food intake, water consumption, and fasting blood glucose (FBG) were recorded weekly for all animals throughout the intervention period. Cage-level consumption was converted to average intake per mouse by dividing the total cage consumption by the number of mice housed in that cage. Fecal samples for SCFA analysis were collected at weeks 0, 4, and 8. Body weight, food intake, water consumption, and fasting blood glucose were recorded weekly. Compartment-specific microbiota profiling, gastrointestinal segment SCFA measurements, histological analyses, and OGTT were performed at the 8-week endpoint.

Before euthanasia, mice were fasted for 12 h. After the 8-week intervention, all animals were euthanized by cervical dislocation, followed by dissection and sample collection. The entire gastrointestinal tract was removed and separated into the stomach, duodenum, jejunum, ileum, cecum, and colon. For standardized sampling, the colon was divided into three equal-length segments, operationally designated as the ascending, transverse, and descending colonic regions for the purposes of this study. Each gastrointestinal segment was opened separately, and the luminal contents were collected into separate sample tubes. Clean sampling instruments were used for each intestinal segment to minimize cross-contamination and carryover. After collection of the luminal contents, the intestinal tissues were washed twice with sterile saline, and the mucosal layer was gently scraped off. The harvested organs, luminal contents, and mucosal samples were snap-frozen in liquid nitrogen and stored at −80 °C for further analysis.

### 2.4. Determination of Fasting Blood Glucose and Oral Glucose Tolerance Test

Mice were fasted for 12 h with free access to water, and FBG was measured weekly via tail vein blood sampling using the glucometer. The oral glucose tolerance test (OGTT) was performed after 8 weeks of MP treatment. Basal blood glucose (0 min) was first measured following a 12 h fast, and a glucose solution (1 g/kg bw) was administered via oral gavage. Blood glucose levels were subsequently recorded at 30, 60, 90, and 120 min to plot the OGTT curve and calculate the corresponding area under the curve [1].

### 2.5. Sequencing and Analysis of Gut Microbiota

Total microbial DNA from fecal samples and from the luminal contents and mucosal samples of the cecum and colon was extracted, quality-assessed, and quantified as previously described by Wang et al. [1]. Small-intestinal microbiota was not profiled in the present study. The bacterial 16S rRNA V3–V4 region was PCR-amplified using primers 338F (5′-barcode+ACTCCTACGGGAGGCAGGCA-3′) and 806R (5′-GGACTACHVGGGTWTCTAAT-3′). Purified and quantified amplicons were used to construct sequencing libraries via the Illumina TruSeq Nano Kit (Illumina, Inc., San Diego, CA, USA), followed by 2 × 250 bp paired end sequencing on the Illumina NovaSeq 6000 platform. Bioinformatics analysis was performed using QIIME2 (2019.4) and R packages (v3.2.0). After primer trimming, sequences were processed via DADA2 for quality control, denoising, and chimera removal to generate amplicon sequence variants (ASVs), which were taxonomically annotated using the Greengenes 13_8 database. Taxonomic composition analysis and Linear discriminant analysis Effect Size (LEfSe) were applied to determine significant structural differences in the gut microbiota and identify key biomarker taxa. The 16S rRNA sequencing was performed in 2022 by Shanghai Personal Biotechnology Co., Ltd. (Shanghai, China).

### 2.6. Histology Analysis

The liver, kidney, pancreas, and intestinal segments (duodenum, jejunum, ileum, cecum, ascending, transverse, and descending colon) of mice were fixed in 4% paraformaldehyde for 24 h. Tissues were dehydrated in a graded alcohol series, embedded by paraffin, cut into 4 μm sections, and baked at 60 °C for drying. Sections were then deparaffinized, stained with hematoxylin and eosin (H&E), and mounted with neutral resin. Histomorphological characteristics were evaluated using an upright optical microscope (NIKON ECLIPSE CI, Nikon Solutions Co., Ltd., Tokyo, Japan) and captured via CaseViewer software (version 2.9, 3DHISTECH Ltd., Budapest, Hungary). H&E-stained sections were evaluated qualitatively to describe general tissue morphology.

### 2.7. Determination of SCFAs

The content of short-chain fatty acids (SCFAs) was determined as previously described by Chen et al. [25]. The collected fecal and gastrointestinal content samples were weighed before sample preparation. Feces and gastrointestinal contents were diluted with distilled water at a ratio of 1:8 (*w*/*v*), mixed thoroughly, and centrifuged at 12,000 rpm and 4 °C for 20 min. SCFA concentrations were normalized to the wet weight of the corresponding fecal or gastrointestinal content sample and are expressed as μmol/g. The supernatant was filtered through a 0.22 μm aqueous membrane filter and analyzed with a gas chromatograph (Agilent 7820A, Agilent Technologies, Inc., Santa Clara, CA, USA) equipped with an AE-FFAP capillary column (30 m × 0.2 mm × 0.25 μm). The inlet and FID detector temperatures were set to 250 °C. The injection volume was 1 μL with a split ratio of 1:12. The flow rates for nitrogen, air, and hydrogen were 30, 400, and 40 mL/min, respectively. The oven temperature program was initiated at 100 °C for 0.5 min, increased to 160 °C at a rate of 4 °C/min, and further increased to 220 °C at 10 °C/min (held for 2 min). Acetic acid, propionic acid, isobutyric acid, and n-butyric acid were quantified using calibration curves established with the corresponding reference standards.

### 2.8. Statistical Analysis

Each mouse was considered an independent biological replicate (*n* = 10 per group). Technical triplicates from the same biological sample were averaged to obtain one value per animal. Data are presented as mean ± standard deviation (SD). Liver and kidney indices and OGTT AUC were analyzed by one-way ANOVA followed by Tukey’s multiple-comparison test. LEfSe was conducted using the default workflow implemented in the MicrobiomeAnalyst platform (https://www.microbiomeanalyst.ca/ (accessed on 4 October 2026)). Unless otherwise specified, *p* < 0.05 was considered statistically significant.

## 3. Results and Discussion

### 3.1. Effects of MPs on Metabolic Parameters in T2DM Mice

**Figure 2:**
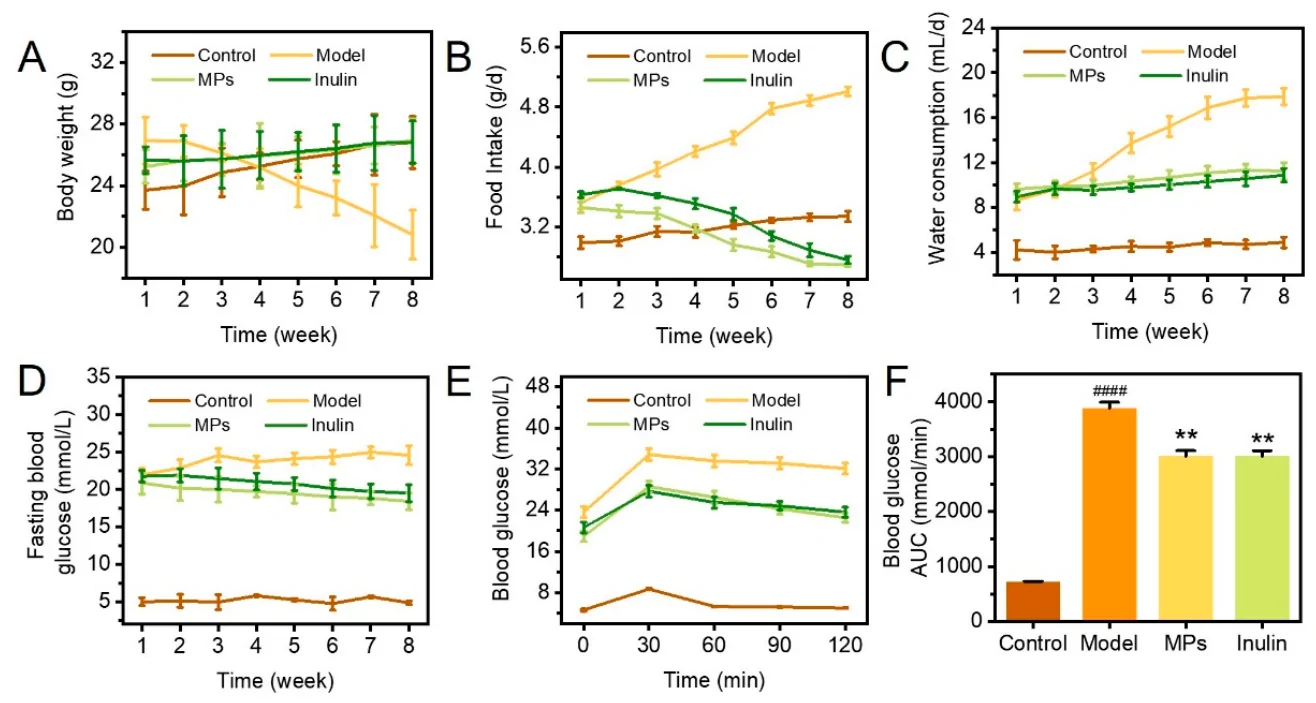
Effect of MP intervention on (**A**) body weight, (**B**) food intake, (**C**) water consumption, (**D**) fasting blood glucose, (**E**) OGTT curve, and (**F**) area under OGTT curve (AUC) of type 2 diabetic mice. Data are presented as mean ± SD. ^####^ *p* < 0.0001 vs. the control group; ** *p* < 0.01 vs. the model group.

As shown in Figure 2A, the body weight of the control group increased by 13.03% after 8 weeks, whereas the model group exhibited a 22.69% decrease, reflecting a typical diabetic symptom. Compared with the model group, MPs and inulin mitigated the weight loss with increases of 6.70% and 4.52%, respectively. Figure 2B,C show an upward trend in daily food and water intake in the model group. Conversely, compared to the model group, MPs and inulin groups exhibited a gradual recovery in food intake and a significant reduction in water consumption by 37.23% and 39.24%, respectively. In terms of glycemic control, compared to the model group, MPs and inulin treatment were associated with the reduction in FBG levels at week 8, with decreases of 25.13% and 20.66%, respectively (Figure 2D), suggesting the hypoglycemic potential of MPs. OGTT results showed that the model group maintained higher blood glucose levels than those of the control group, indicating impaired glucose tolerance in T2DM mice. Notably, MP and inulin treatment led to a significant downward shift in the OGTT curves (Figure 2E). The corresponding AUC values of the MP (3004.33 ± 104.92 mmol/min) and inulin (3007.00 ± 100.43 mmol/min) groups were significantly lower than that of the model group (3881.67 ± 110.64 mmol/min, *p* < 0.01, Figure 2F). The results collectively suggested that MPs effectively ameliorated glucose intolerance in T2DM mice, which was consistent with the results reported by Li et al. [12].

### 3.2. Effect of MPs on Organ Morphology and Structural Integrity in T2DM Mice

**Figure 3:**
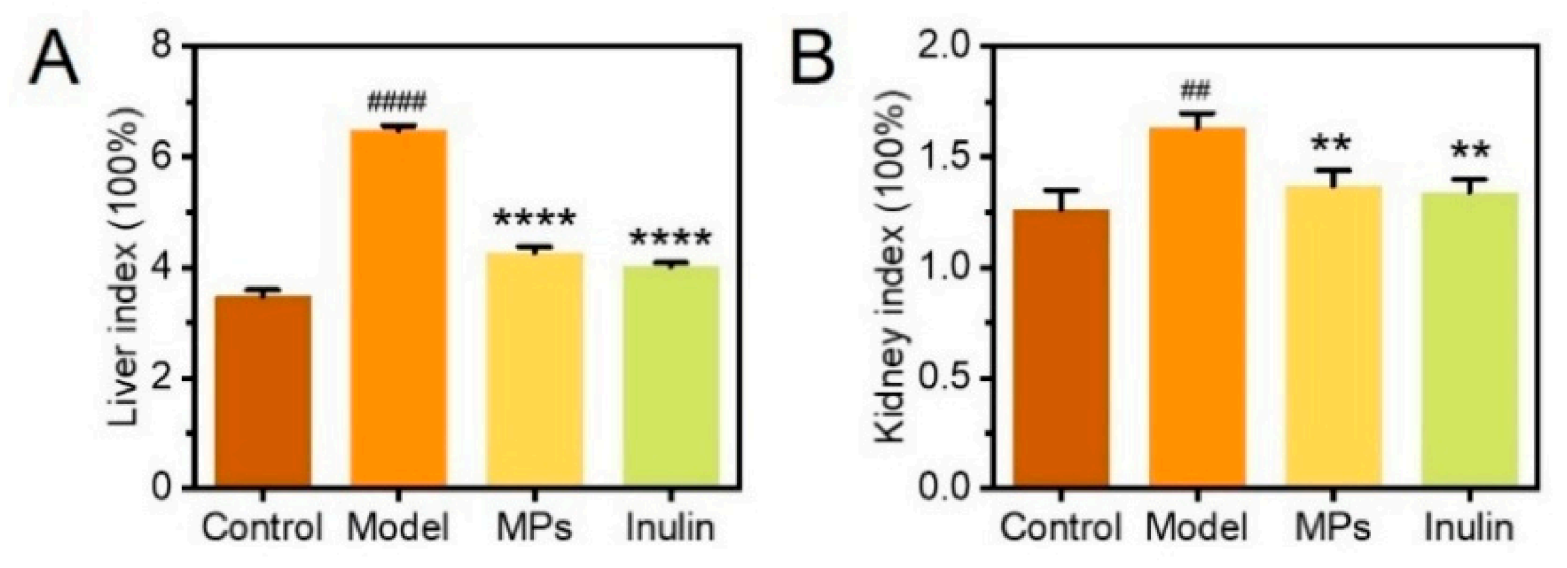
Effect of MPs on (**A**) liver index and (**B**) kidney index in T2DM. Data are presented as mean ± SD. ^##^ *p* < 0.01, and ^####^ *p* < 0.0001 vs. the control group; ** *p* < 0.01, and **** *p* < 0.0001 vs. the model group.

The liver and kidney indices and the histomorphological changes in the primary metabolic organ (pancreas) and the entire intestinal tract (duodenum, jejunum, ileum, cecum, and colon) were analyzed to further evaluate the protective effects of MPs against tissue metabolic dysfunction and organ damage in T2DM mice. The liver and kidney indices were determined to evaluate changes in relative organ weights in T2DM mice. As shown in Figure 3A, the liver index of the model group significantly increased by 46.45% compared with the control group (*p* < 0.05), whereas it decreased by 34.10% and 38.11% in the MP and inulin groups, respectively. Similarly, the kidney index increased by 29.37% in the model group compared with the control group (*p* < 0.05), while it decreased by 15.95% and 17.79% in the MP and inulin groups, respectively (Figure 3B). These results indicate that MP intervention was associated with changes in relative liver and kidney weights in T2DM mice.

**Figure 4:**
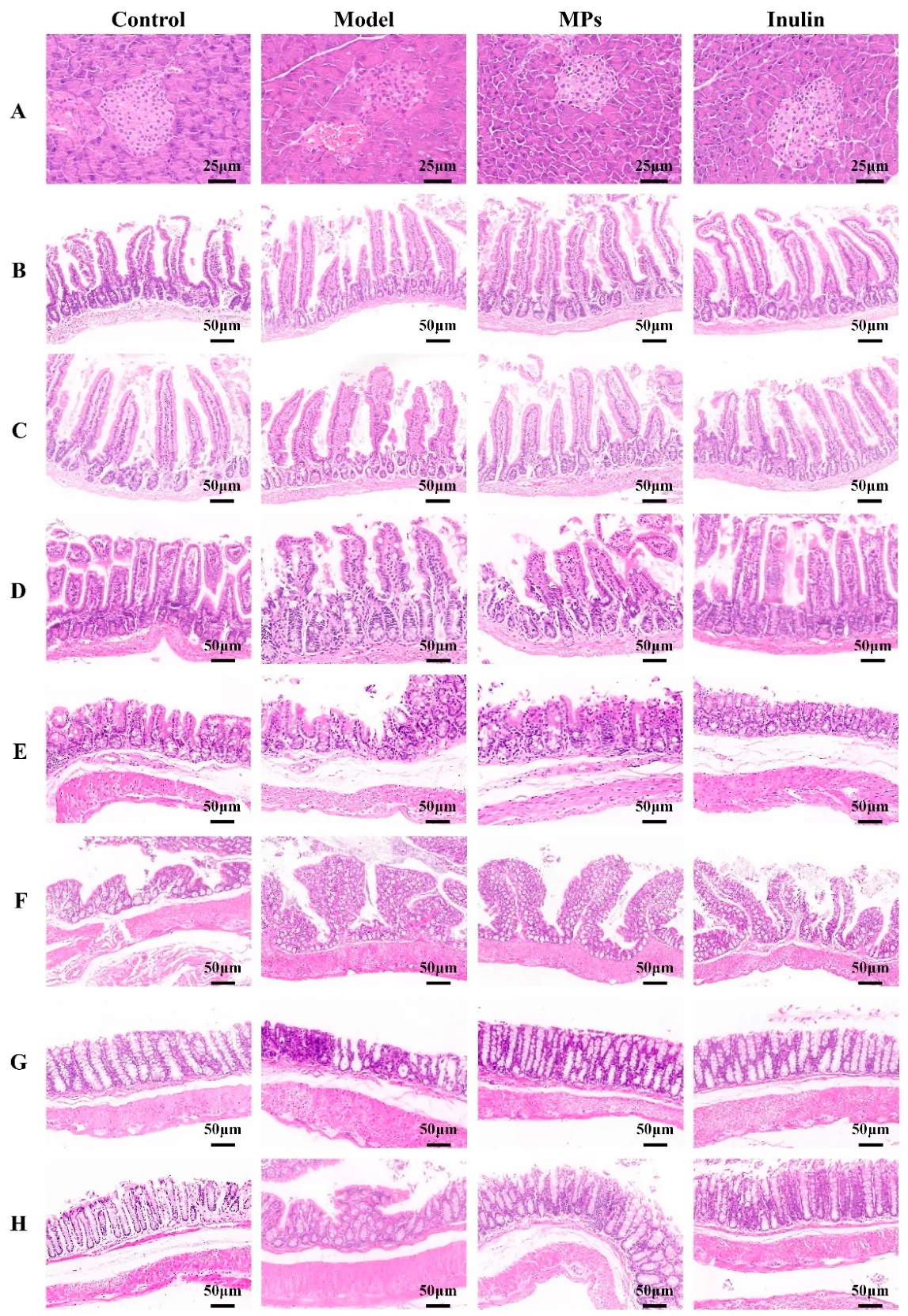
Representative H&E-stained sections showing the morphology of the (**A**) pancreas, (**B**) duodenum, (**C**) jejunum, (**D**) ileum, (**E**) cecum, (**F**) ascending colon, (**G**) transverse colon, and (**H**) descending colon in T2DM mice. Images are presented for qualitative histomorphological assessment. Hematoxylin stains nuclei blue to purple, whereas eosin stains the cytoplasm and extracellular matrix pink.

Pancreatic islet function plays a crucial role in maintaining overall metabolic homeostasis and glycemic control mediated by insulin [26]. The histopathological results of the pancreas showed that the islets in the control group exhibited an intact structure and clear boundaries, with β-cells displaying regular morphology and uniform distribution (Figure 4A). In contrast, the model group showed reduced islet volume, impaired structural integrity, and blurred boundaries. The β-cells also presented pathological changes including pyknosis and karyolysis, which was consistent with a previous report on T2DM mice [27]. The MP- and inulin-treated groups showed less pronounced pancreatic histomorphological alterations than the model group, including apparently reduced islet atrophy and improved structural integrity. These results indicated that MPs may have a reparative effect on pancreatic islet structure and β-cell integrity.

The morphological and structural integrity of the intestine is fundamental to maintaining metabolic homeostasis, whereas diabetes frequently leads to intestinal structural damage and inflammatory responses. The morphological changes across the entire intestinal tract (from the duodenum to the colon) in T2DM mice treated with MPs and inulin are illustrated in Figure 4B–H. The intestinal tissue of the mice in the control group was normal, with clear and intact cells in the lamina propria of the mucosa. The villi were elongated, densely packed, and structurally intact. The submucosal tissue was dense, and the crypts exhibited a regular tubular shape with a near arrangement. The epithelial cells showed no obvious degeneration or shedding, and no significant pathological changes or inflammatory infiltration were observed. In contrast, the intestinal tissues of the model mice exhibited significant pathological damage, primarily characterized by the disruption of tissue architecture, irregular arrangement of the mucosal layer, and distorted, disorganized crypts. Furthermore, erosion and shedding of villous epithelial cells, along with the necrosis and disintegration of crypt epithelial cells, were observed, accompanied by severe pyknosis and inflammatory cell infiltration. The MP- and inulin-treated groups showed less pronounced mucosal architectural disruption than the model group, with comparatively better-preserved villus and crypt morphology. Both pyknosis and inflammatory responses were also markedly alleviated. Taken together, the improvements in organ indices and histomorphology across the pancreas and intestinal tract suggested a broader amelioration of diabetes-associated tissue alterations following MP administration.

### 3.3. SCFA Concentrations in Feces and Different Gastrointestinal Segments

#### 3.3.1. Temporal Changes in Fecal SCFA Concentrations

**Figure 5:**
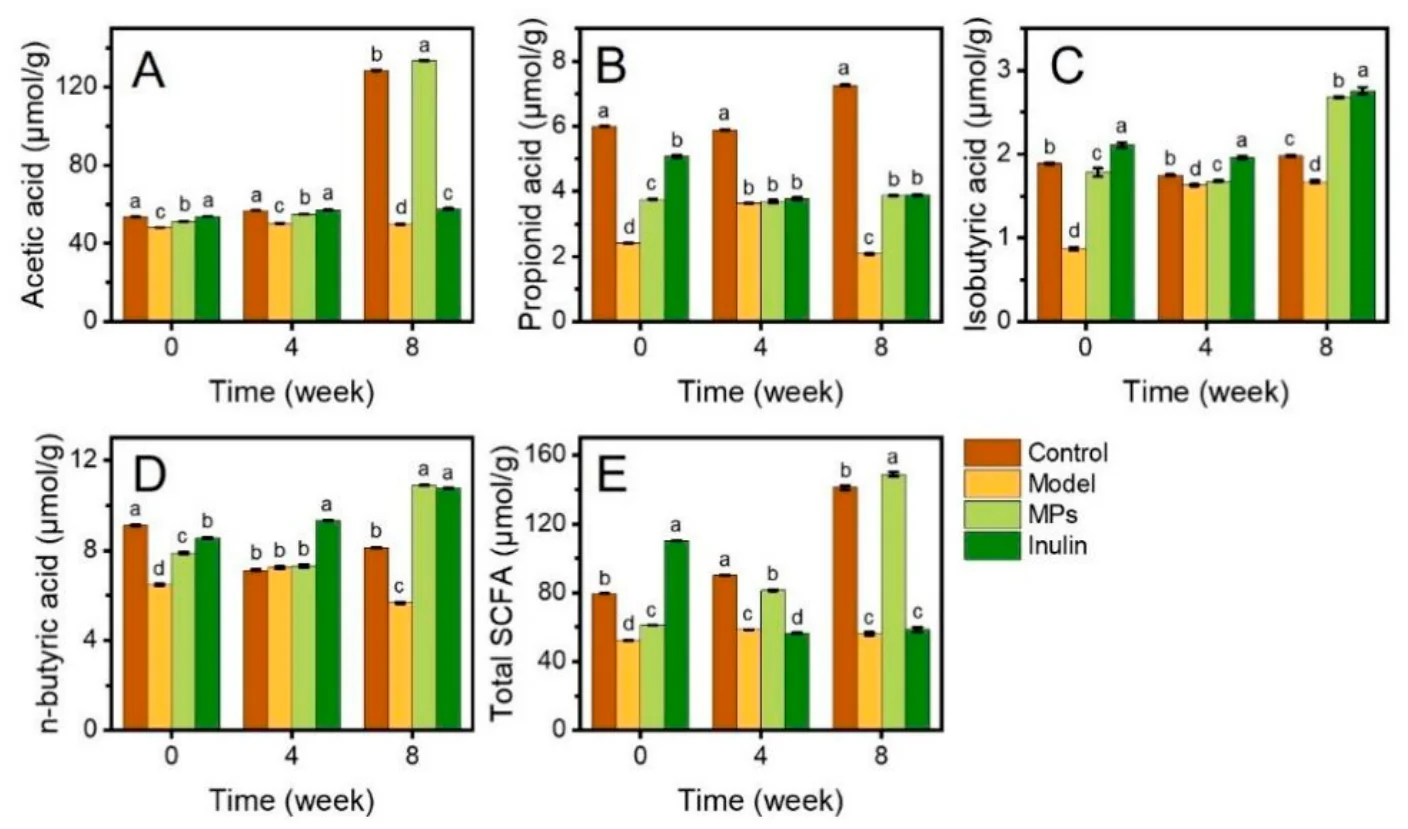
Effects of the MP intervention on the content of (**A**) acetic acid, (**B**) propionic acid, (**C**) isobutyric acid, (**D**) n-butyric acid, and (**E**) total SCFA in the feces of T2DM at weeks 0, 4, and 8. Different letters indicate significant difference (*p* < 0.05).

Figure 5 illustrates the temporal variations in fecal SCFA concentrations in T2DM mice at weeks 0, 4, and 8 of the MP intervention. Compared with the control group, the fecal contents of acetic acid, propionic acid, isobutyric acid, and n-butyric acid in the model mice significantly decreased by 61.26%, 71.35%, 15.66%, and 30.17%, respectively (*p* < 0.05), indicating the dysregulation of intestinal SCFA profiles under T2DM conditions. After 4 weeks of MP administration, the fecal contents of acetic acid and isobutyric acid significantly increased to 55.02 and 1.68 μmol/g, compared to 50.31 and 1.63 μmol/g in the model group. Moreover, the total SCFA content was significantly elevated by 39.11% compared with the model group (*p* < 0.05). As the intervention period extended to 8 weeks, SCFA levels further increased. In the MP group, the contents of acetic acid, propionic acid, isobutyric acid, n-butyric acid, and total SCFAs significantly increased to 133.66, 3.87, 2.68, 10.89, and 148.87 μmol/g, respectively, from 49.87, 2.08, 1.67, 5.67, and 56.23 μmol/g in the model group (*p* < 0.05). At week 8, the levels of acetic acid, isobutyric acid, n-butyric acid, and total SCFAs in the MP group exceeded those of the control group by 3.84%, 35.35%, 34.11%, and 5.41%, respectively. These elevations were considered intervention-associated changes in SCFA profiles. This indicated that MP administration was associated with time-dependent changes in fecal SCFA concentrations in T2DM mice.

#### 3.3.2. Spatial Distribution of SCFAs Across Gastrointestinal Segments

**Figure 6:**
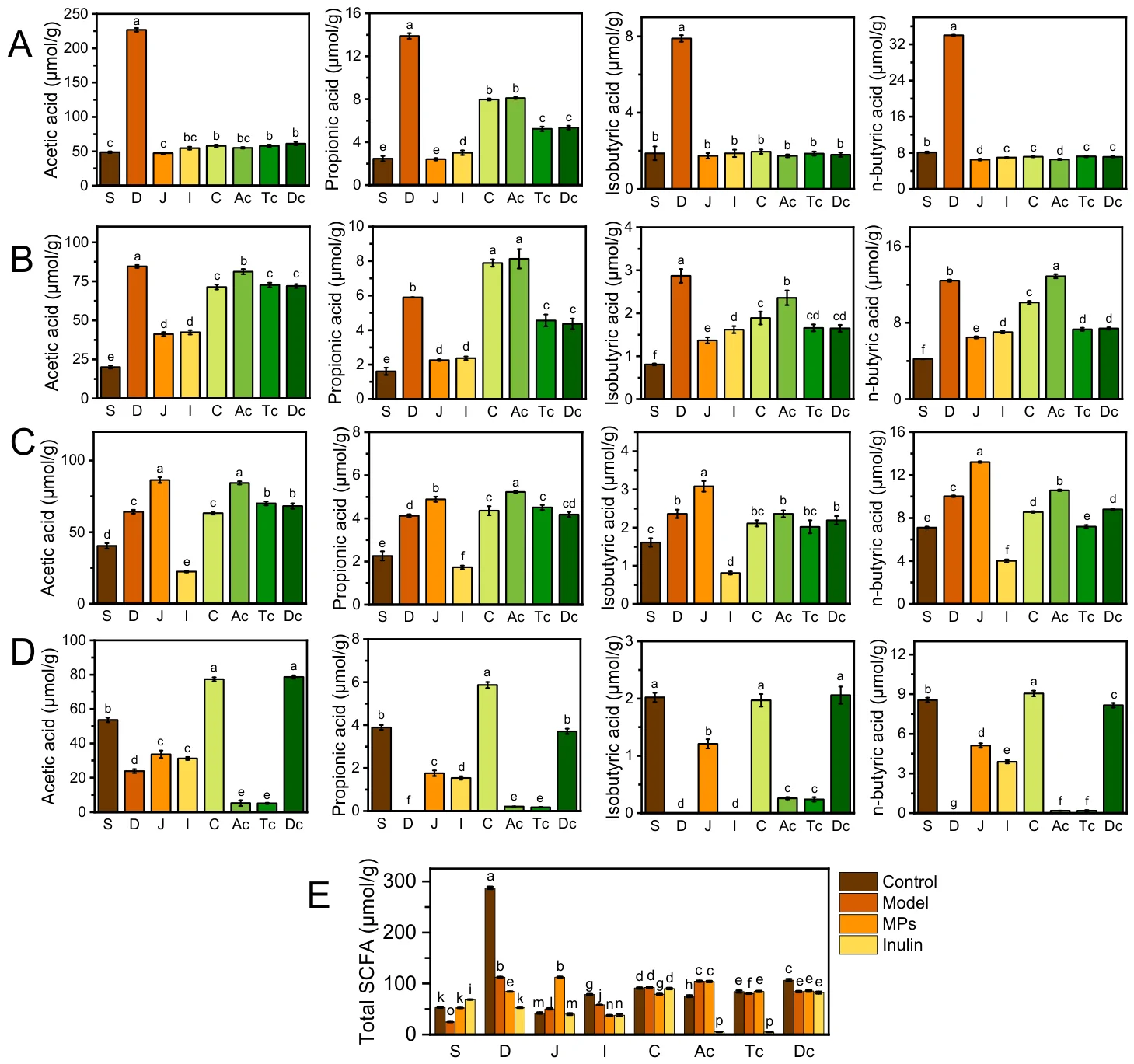
SCFA concentrations in different gastrointestinal segments including the stomach (S), duodenum (D), jejunum (J), ileum (I), cecum (C), ascending colon (Ac), transverse colon (Tc), and descending colon (Dc) of T2DM in the (**A**) control group, (**B**) model group, (**C**) MP group, and (**D**) inulin group. (**E**) Total SCFA contents in different gastrointestinal segments of T2DM treated with MPs. Different letters indicate significant difference (*p* < 0.05).

To further explore the potential spatial distribution patterns associated with MP intervention, the SCFA levels across different gastrointestinal segments in mice from the control, model, MP, and inulin groups were further analyzed. As shown in Figure 6A, the duodenum of the control mice exhibited the highest contents of acetic acid (226.89 μmol/g), propionic acid (13.89 μmol/g), isobutyric acid (7.89 μmol/g), and n-butyric acid (34.01 μmol/g). The relatively high SCFA concentrations observed in the duodenum may reflect multiple physiological and metabolic factors. However, the present study did not determine the specific sources or production sites of these SCFAs. Compared with the control group, the isobutyric acid levels in the stomach, duodenum, jejunum, and transverse colon of the model group decreased by 56.68%, 63.62%, 21.26%, and 10.75%, respectively. Meanwhile, n-butyric acid levels in the stomach and duodenum fell by 47.91% and 63.48%, respectively. This indicated that T2DM significantly impaired n-butyric acid and isobutyric acid levels across multiple gastrointestinal segments, both of which are crucial for regulating host glucose metabolism, improving insulin sensitivity, and inhibiting hepatic gluconeogenesis [28].

After week 8 of the MP intervention, propionic acid levels in the stomach and jejunum of T2DM mice recovered to 2.26 and 4.89 μmol/g, increasing significantly by 40.37% and 116.37% compared to the model group (*p* < 0.05). Moreover, compared to the model group, isobutyric acid levels in the stomach, jejunum, transverse colon, and descending colon were elevated by 98.77%, 124.82%, 17.82%, and 32.73%, respectively, while n-butyric acid levels in the stomach, jejunum, and descending colon rose by 68.09%, 103.86%, and 18.89%. It can be seen that MP intervention effectively increased n-butyric acid and isobutyric acid concentrations across multiple intestinal segments. As the primary energy source for colonocytes, n-butyric acid has been reported to contribute to maintaining the mucosal barrier and promoting sodium and water absorption. Furthermore, butyrate regulates colonic motility, increases local blood flow, and prevents inflammation, which may contribute to ameliorating T2DM pathology [1,29].

Additionally, compared with the control group, the SCFA levels in the stomach, duodenum, ileum, transverse colon, and descending colon of the model mice significantly decreased by 53.45%, 60.90%, 25.80%, 4.78%, and 20.91%, respectively. However, MP intervention significantly increased the total SCFA levels in the stomach, jejunum, and transverse colon by 110.68%, 122.98%, and 5.27%, respectively, compared to the model group. These results suggested that MPs were associated with alterations in total SCFA concentrations across different intestinal segments in T2DM mice.

### 3.4. Spatial Distribution of Cecal and Colonic Microbiota

#### 3.4.1. Spatial Distribution of Cecal and Colonic Microbiota at the Phylum Level

**Figure 7:**
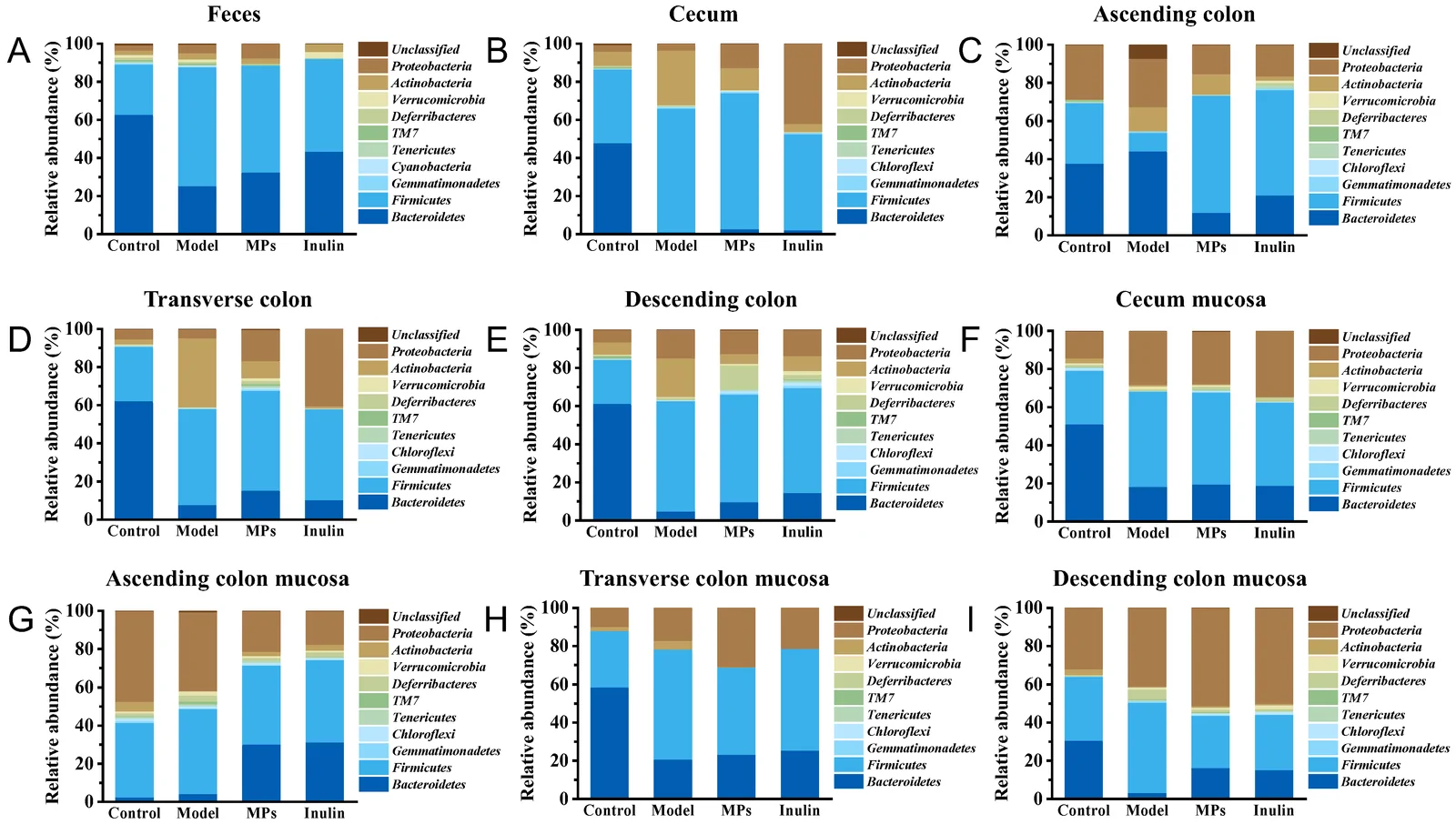
Effects of MPs on microbiota composition in fecal, cecal, and colonic samples at the phylum level in T2DM mice: (**A**) feces; (**B**) cecum; (**C**) ascending colon; (**D**) transverse colon; (**E**) descending colon; (**F**) cecum mucosa; (**G**) ascending colon mucosa; (**H**) transverse colon mucosa; and (**I**) descending colon mucosa.

To investigate the spatial distribution patterns of gut microbiota in T2DM mice, the microbial composition at the phylum level of the intestinal tissues (contents and mucosa of the cecum and colon) was analyzed after an 8-week MP intervention. As illustrated in Figure 7, Bacteroidetes, Firmicutes, Actinobacteria, and Proteobacteria were the predominant phyla with the highest relative abundances in the intestinal contents across the mice in control, model, MP, and inulin groups. A similar gut microbiota composition of T2DM mice can be observed in an existing report [1]. The T2DM mice exhibited increased Firmicutes relative abundance but decreased Bacteroidetes relative abundance. A similar trend has also been reported in male C57BL/6J mice induced by a high-fat diet [30,31].

Figure 7 shows the phylum-level taxonomic composition of the gut microbiota across the cecal and colonic contents and mucosa in the four experimental groups. In the control group, fecal microbiota was primarily dominated by Bacteroidetes and Firmicutes, with average relative abundances of 62.70% and 26.42%, respectively (Figure 7A). Furthermore, Proteobacteria emerged as a major dominant phylum in specific localized intestinal segments, such as the contents and mucosa of the ascending colon and the mucosa of the descending colon. Conversely, T2DM mice exhibited a reduction in Bacteroidetes across most sampled cecal and colonic compartments, paralleled by an elevated relative abundance of Firmicutes. With the alteration in core commensal gut microbiota, opportunistic phyla showed increased relative abundance, highlighted by the enrichment of Proteobacteria and Actinobacteria. Compared to the control group, Proteobacteria notably increased by 13.86%, 6.18%, 8.62%, and 9.11% in the cecal mucosa, transverse colon mucosa, and the contents and mucosa of the descending colon, respectively. Simultaneously, the relative abundance of Actinobacteria in the contents of the cecum, ascending colon, transverse colon, and descending colon experienced a dramatic surge from 7.63%, 0.03%, 2.64%, and 6.35% to 28.69%, 12.61%, 36.21%, and 14.97%, respectively.

To characterize the gut microbiota alterations associated with T2DM, MP intervention was associated with an increase in the relative abundance of Bacteroidetes, which was reduced in the model group. Specifically, the relative abundance of Bacteroidetes in feces increased by 7.16% compared with the model group, indicating a fecal microbial response to MP intervention. Spatially, this restorative effect was particularly pronounced across various colonic segments, especially within the mucosal layer. Specifically, its relative abundance increased by 1.58%, 7.37%, and 4.83% in the contents of the cecum, transverse colon, and descending colon, respectively, while achieving substantial recoveries of 25.92%, 2.82%, and 13.08% in the mucosa of the ascending, transverse, and descending colon. Conversely, for Actinobacteria, which showed an increased relative abundance under T2DM conditions, MP intervention was associated with lower relative abundance by 17.03%, 27.24%, and 15.08% in the contents of the cecum, transverse colon, and descending colon, respectively, alongside a 3.91% downregulation in the transverse colon mucosa. Taxonomically, the phylum Actinobacteria exhibited high heterogeneity. It encompassed beneficial bacteria such as *Bifidobacterium* [32], whereas its increased relative abundance in metabolic disease models has been associated with shifts in specific opportunistic microbial populations under disease-associated intestinal environments [33]. The MP-associated reduction in Actinobacteria relative abundance suggested that MPs may contribute to the reshaping of localized microbial community structures, potentially altering the ecological conditions that favor the enrichment of disease-associated taxa. Furthermore, the increased relative abundance of Proteobacteria in T2DM mice did not experience uniform suppression with MP intervention. Instead, it exhibited profound spatial heterogeneity. This was evidenced by reductions of 10.04%, 2.74%, and 19.66% in the ascending colon contents, descending colon contents, and ascending colon mucosa, respectively. In contrast, it showed elevations of 9.33%, 11.45%, 13.15%, and 10.00% in the cecum, the transverse colonic contents and mucosa, and the descending colonic mucosa, respectively. The phylum Proteobacteria is highly diverse, encompassing not only lipopolysaccharide-rich pro-inflammatory pathogens but also commensal bacteria involved in complex biochemical pathways [34]. The MP-associated elevation of Proteobacteria in certain intestinal segments may not necessarily indicate an increase in pathogenic taxa. Rather, it may reflect changes in community composition associated with alterations in dominant taxa [35], such as Bacteroidetes. This intricate phenomenon suggested the complex and heterogeneous patterns of microbial community responses following MP intervention. Species-level microbe–metabolite associations may provide greater functional insight than broad phylum-level changes [36].

In terms of the overall regulatory results, MPs exhibited prebiotic-like microbial modulation characteristics that were broadly consistent with those observed in the inulin group, which was included as a prebiotic comparator. Recent evidence further indicates that the anti-diabetic effects of polysaccharides are structure-dependent and may involve gut microbiota modulation [37]. Across the contents of the cecum and the ascending, transverse, and descending colon, as well as the mucosa of the ascending, transverse, and descending colon, MPs and inulin demonstrated highly congruent trajectories in reducing the increased relative abundance of Proteobacteria and Actinobacteria and modulating the composition of commensal gut microbiota. This similarity implied that MPs may be preferentially associated with specific microbial taxa within the cecum and colon, potentially influencing the local metabolic environment and favoring the enrichment of beneficial microbial populations while reducing the relative abundance of disease-associated taxa. Notably, the efficacy of MPs in restoring Bacteroidetes and reducing the increased relative abundance of specific phyla even exhibited a trend superior to that of inulin in critical intestinal segments such as the transverse colonic contents and the descending colonic mucosa. This further supported its potential role as a modulator of gut microbial ecology. The microbial compositional patterns will be further characterized in the subsequent genus-level analysis.

#### 3.4.2. Spatial Heterogeneity of Gut Microbiota at the Genus Level

**Figure 8:**
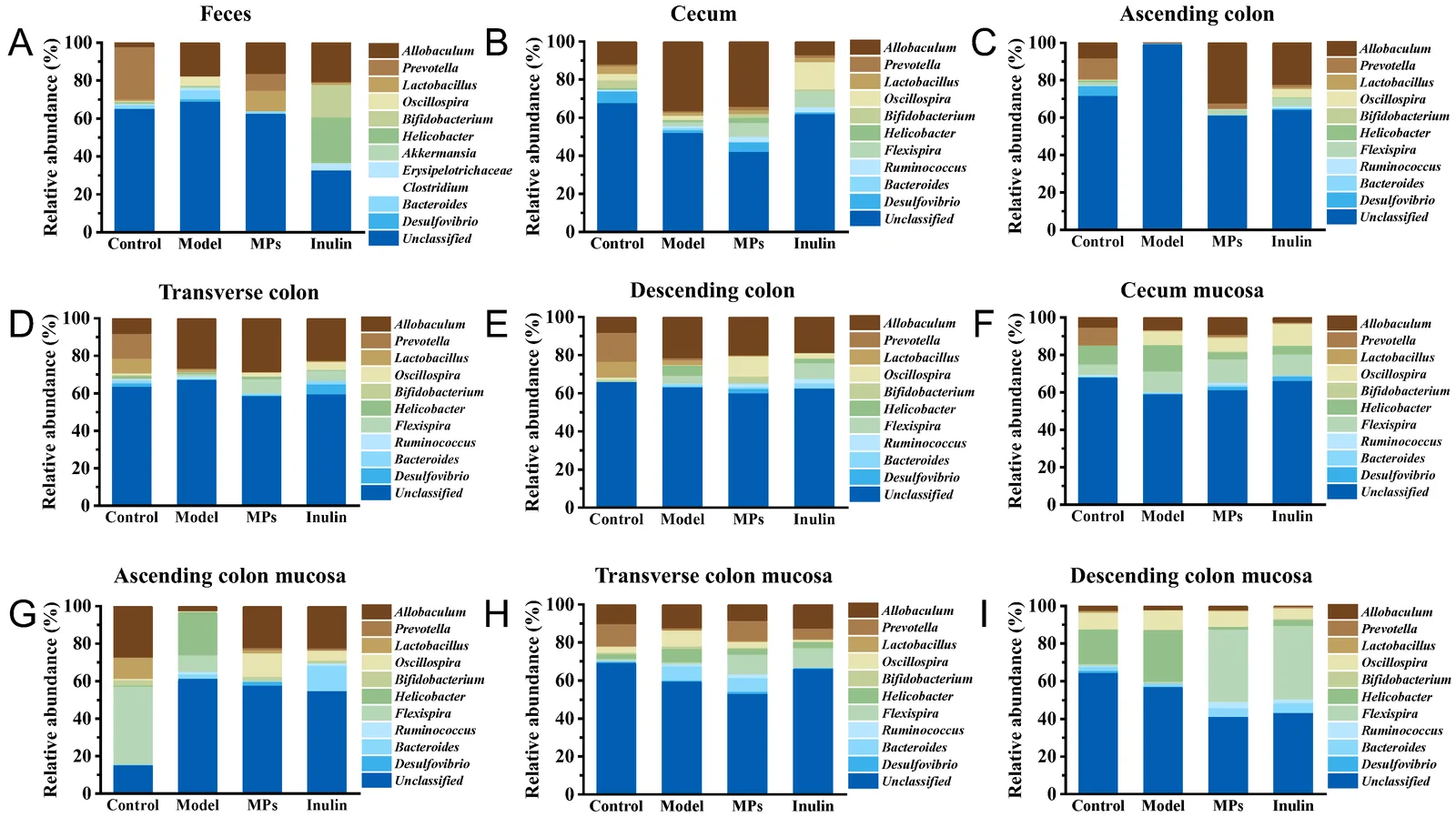
Effects of MPs on microbiota composition in fecal, cecal, and colonic samples at the genus level in T2DM mice: (**A**) feces; (**B**) cecum; (**C**) ascending colon; (**D**) transverse colon; (**E**) descending colon; (**F**) cecum mucosa; (**G**) ascending colon mucosa; (**H**) transverse colon mucosa; and (**I**) descending colon mucosa. The ‘Unclassified’ category represents sequences that could not be assigned at the genus level.

The cecal and colonic microbiota at the genus level exhibited profound spatial heterogeneity with MP intervention. As a reported SCFA-associated bacterial genus in the gut, *Allobaculum* demonstrated distinct spatial distribution patterns under T2DM conditions [38]. As illustrated in Figure 8, *Allobaculum*, a representative fermentative bacterial genus, displayed spatial alterations in its distribution patterns under T2DM conditions. In the cecum, an important fermentation compartment in mice, the relative abundance of *Allobaculum* in the model group increased by 24.49% compared with the control group. In the ascending colonic contents of the model group, the relative abundance of *Allobaculum* decreased to 0.12%, while 99.56% of the genus-level sequences were classified as unclassified taxa. Given this high proportion of unclassified sequences, genus-level taxonomic resolution in this compartment was limited, and no specific ecological mechanism was inferred from this pattern.

Notably, MPs and inulin interventions were associated with distinct spatial changes in the relative abundance of *Allobaculum*. Compared with the model group, MPs increased the relative abundance of *Allobaculum* in the ascending colonic lumen, whereas its relative abundance in the transverse colon and descending colonic mucosa shifted toward levels observed in the control group. These findings describe compartment-specific compositional changes associated with MP intervention but do not establish the ecological processes underlying these patterns.

The genus *Helicobacter* has been reported as an inflammation-associated bacterial group [39], which exhibits a high degree of mucosal colonization preference [40]. The relative abundance of this bacterium remained at low levels across the samples from the cecal and colonic mucosal compartments. However, an increase in its relative abundance was observed within the mucosal layer closely adhering to the intestinal wall in the T2DM model. During T2DM progression, prolonged hyperglycemic stress and lipid metabolism disorders have been reported to affect intestinal barrier-related functions, including mucus layer integrity and local oxidative stress status [41]. This altered mucosal barrier environment may provide favorable conditions for the increased relative abundance of *Helicobacter*. Compared to the control group, the relative abundance of *Helicobacter* in the mucosal layers of the cecum, ascending colon, transverse colon, and descending colon in T2DM model mice increased by 4.68%, 22.80%, 4.87%, and 8.94%, respectively. The increased abundance of mucosa-associated inflammation-related bacteria has been reported to be associated with alterations in mucus barrier integrity and inflammatory responses, which may contribute to systemic metabolic disturbances [42]. Notably, MP intervention demonstrated a potential modulatory effect on mucosa-associated microbial communities. The administration of MPs reduced the relative abundance of *Helicobacter* within the mucus-associated microbial communities across the fermentative intestinal segments, resulting in decreased relative abundance in the mucosal layers of the cecum, ascending colon, transverse colon, and descending colon of T2DM mice by 10.20%, 23.17%, 4.11%, and 26.00%, respectively. Furthermore, compared with the control group, MP intervention also decreased the relative abundance of *Helicobacter* in the cecum and descending colonic mucosa by 5.52% and 17.00%, respectively.

Interestingly, the results revealed that the genus *Flexispira*, which is taxonomically within the same family as *Helicobacter* [43], exhibited a distinct spatial pattern of relative abundance in both the contents and mucosal layers that was opposite to that of the latter. The relative abundance of *Flexispira* was lower in the T2DM model group. Its relative abundance was restored following MP intervention in the contents of the cecum, ascending colon, and transverse colon after MP intervention, as well as in the mucosal layers of the transverse and descending colon, by 5.00%, 3.24%, 6.43%, and 37.36%, respectively. This characteristic shift in relative abundance is highly consistent with the regulatory trend of the classic prebiotic inulin, suggesting that the increased abundance of this commensal genus may be associated with microbial community changes related to intestinal ecological balance. The inverse abundance dynamics observed at the gut microecological interface suggested potential niche differentiation or distinct ecological responses between related taxa within the same taxonomic family.

As a key genus associated with the degradation of complex plant polymers within the gut microbiota, *Prevotella* exhibited substrate-responsive distribution patterns [44]. In the control group, *Prevotella* maintained a stable relative abundance across the sampled cecal and colonic compartments, presenting as one of the dominant taxa in the contents of the ascending colon (11.36%), transverse colon (13.26%), and descending colon (15.23%), as well as in the mucosa of the cecum (8.56%) and transverse colon (11.56%). Conversely, in the T2DM model group, the relative abundance of this bacterium was reduced across multiple samples cecal and colonic compartments, indicating an overall decrease in its distribution under T2DM conditions. This decrease may not simply reflect reduced nutrient availability. Rather, it may be related to changes in the availability of microbiota-accessible carbohydrates and other ecological factors under high-fat/high-sugar diet conditions, which may influence the competitive position of this genus within the gut community. This ecological interpretation may also be applicable to the MPs and inulin groups subjected under the T2DM modeling condition. Nevertheless, compared to the model group, MPs partially restored the relative abundance of *Prevotella* by 3.01%, 0.68%, 1.04%, and 9.35% in the ascending colonic contents, cecal mucosa, ascending colonic mucosa, and transverse colonic mucosa, respectively. This was consistent with the trend observed in the mice with inulin intervention. MP intervention induced a marked alteration in the relative abundance of *Prevotella* within the transverse colonic mucosa. Given that *Prevotella* possessed the capacity to degrade non-structural carbohydrates [45], the restoration of its abundance suggested that MPs may exhibit prebiotic-like effects by modulating carbohydrate-associated microbial populations within the intestine. The restored *Prevotella* may contribute to carbohydrate-associated microbial metabolism and SCFA-related metabolic processes, which is consistent with our prior discussion on SCFAs.

*Oscillospira* was a low-abundance and butyrate-producing genus within the gut microbiota [46], which exhibited a distinct abundance shift potentially associated with altered substrate availability under T2DM conditions. The results showed that the overall abundance of *Oscillospira* in the control group remained at a relatively low and stable level. However, the spatial distribution of *Oscillospira* exhibited marked compartment-specific differences in the T2DM model group. Its relative abundance was markedly reduced in the luminal contents across the sampled cecal and colonic compartments. Conversely, within the mucosal layer, particularly in the distal transverse colonic mucosa (8.36%) and descending colonic mucosa (10.23%), its relative abundance increased markedly and reached the highest levels among all groups. This striking dichotomy of reduced abundance in the intestinal lumen coupled with increased abundance in the distal mucosa may reflect compartment-specific microbial responses associated with altered substrate conditions within the gut. Under conditions of limited carbon availability, commensal bacteria have been reported to exhibit metabolic adaptations that may involve utilization of alternative host-derived substrates such as mucins [47]. Particularly in the distal colon, the increased relative abundance of *Oscillospira* within the mucosal layer may represent an adaptive microbial response to altered ecological conditions, potentially involving shifts in substrate utilization strategies. In contrast, MPs and inulin interventions increased the relative abundance of *Oscillospira* in the contents of certain segments and the proximal (cecal and ascending colonic) mucosa. This increase in relative abundance suggested a potential recovery of the ecological distribution of this genus within the sampled cecal and colonic compartments, which may be related to butyrate-associated microbial functions. Additionally, MPs and inulin interventions may have modified the ecological conditions within distal intestinal segments, potentially contributing to the reduction in the increased relative abundance of this bacterium in the transverse and descending colonic mucosa.

Overall, MPs induced spatial alterations in gut microbial community structure and SCFA-associated metabolic patterns, which were accompanied by improvements in metabolic phenotypes. Within the luminal contents, MPs exhibited prebiotic-like microbial modulation characteristics, which were associated with the restoration of the relative abundance patterns of *Prevotella* and *Oscillospira*, while reducing the increased relative abundance of *Allobaculum* and altering microbial distribution patterns across the sampled cecal and colonic compartments. Within the mucosal layer, MPs were associated with reduced relative abundance of the inflammation-associated genus *Helicobacter* and increased representation of *Flexispira*, suggesting potential shifts in mucosa-associated microbial ecological patterns. These multidimensional spatial microbial alterations occurred alongside improved metabolic outcomes. However, the present data do not establish the direction of this relationship. The microbial changes may act as causal mediators, arise in response to altered host physiology or luminal conditions, occur as parallel consequences of MP intervention, or reflect a combination of these processes.

### 3.5. Spatiotemporal Microecological Alterations Associated with MP Intervention in T2DM

**Figure 9:**
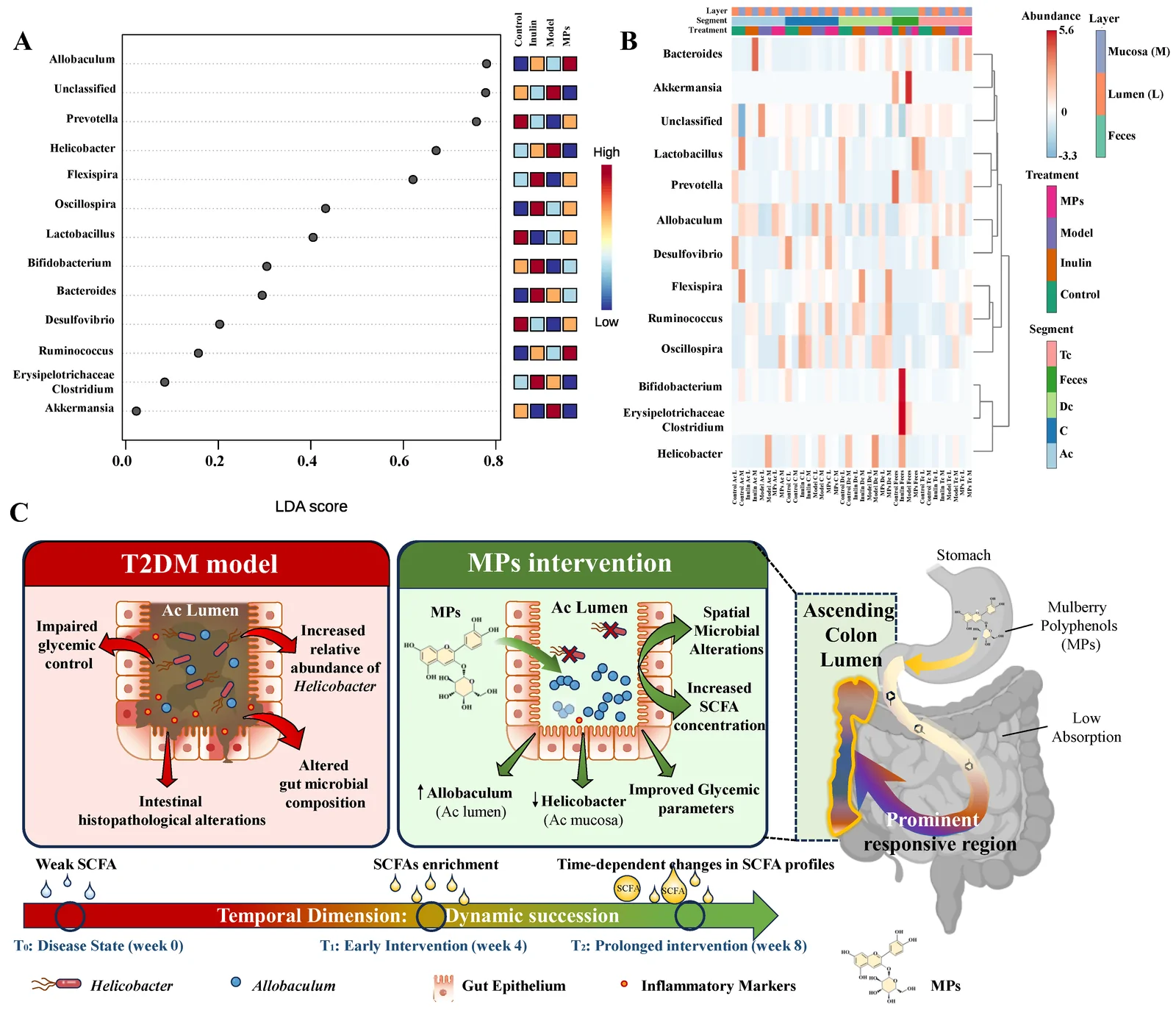
(**A**) LDA scores and (**B**) LEfSe analysis yielded information on taxonomic units with significant differences between different sites of type 2 diabetic mice. Cecum (**C**) and ascending colon contents (Ac), transverse colon contents (Tc), and descending colon contents (Dc). Different arrow colors are used only for visual distinction and have no specific biological meaning.

Based on the integrated analyses, we summarized the spatial microbial patterns associated with MP intervention (Figure 9). LEfSe analysis (Figure 8A) identified *Helicobacter* as a disease-associated discriminatory genus in the T2DM model, whereas MP intervention was associated with an increased relative abundance of *Allobaculum*, which showed a high LDA score. Spatial mapping (Figure 8B) showed that these microbial changes were heterogeneous across the sampled cecal and colonic compartments and were particularly pronounced in the ascending-colon region. These observations support the hypothesis that the ascending-colon region may represent an important responsive compartment to MP intervention, but they do not establish an ascending-colon-anchored mechanism.

Temporally, the spatially resolved microbial alterations in the ascending colon were accompanied by time-dependent changes in fecal SCFA profiles (Figure 8C). From the initial disease state (Week 0) through early intervention (Week 4) to prolonged intervention (Week 8), the progressive increase in SCFA levels, particularly acetate, butyrate, and isobutyrate, was observed alongside improvements in metabolic phenotypes. Although these SCFAs have reported roles in colonocyte metabolism and intestinal homeostasis, the present study did not directly test whether these changes mediated the metabolic effects of MPs. Importantly, SCFA levels exceeding those of the controls should not necessarily be interpreted as beneficial, because their physiological effects depend on SCFA species, concentration, anatomical location, absorption, and host metabolic context. Overall, MP intervention was associated with spatial changes in cecal and colonic microbial composition and time-dependent changes in fecal SCFA profiles.

Collectively, the multidimensional spatiotemporal model, integrating spatial alterations in proximal colon-associated bacterial communities with temporal changes in SCFA profiles, provides a potential framework linking microbial ecological modulation with improved metabolic phenotypes in T2DM. These findings provide new insights into polyphenol–gut microbiota interactions from a spatiotemporal perspective and provide a basis for future mechanistic and translational studies in T2DM.

Although the HFD/STZ-induced T2DM mouse model used in this study incorporates both dietary metabolic stress and partial β-cell impairment, it does not completely reproduce the multifactorial pathogenesis of human T2DM, particularly obesity-associated insulin resistance and genetic susceptibility [48]. Similarly, the 500 mg/kg bw dose of MPs used in the present study represents an experimental dose selected for a preclinical mouse model and should not be directly extrapolated to human dietary intake or therapeutic dosing. Further dose–response studies, pharmacokinetic characterization, and clinical validation are required to determine the translational relevance and appropriate intake levels of MPs in humans. In addition, inulin was included as a prebiotic comparator rather than a conventional pharmacological positive control, and no standard anti-diabetic-drug group was included. Therefore, the effects of MPs cannot be directly compared with those of established pharmacological treatments. Moreover, the equal-length colonic subdivisions used in this study represent operational sampling regions rather than anatomically defined regions directly comparable with human colonic segments. Gastrointestinal compartmentalization and microbial ecology differ substantially between mice and humans. In particular, murine coprophagy can increase recurrent microbial exposure in the proximal gastrointestinal tract [49], while SPF housing can further influence microbial community composition [50]. Because small-intestinal microbiota was not profiled in the present study, our findings do not address persistent small-intestinal colonization or local microbial fermentation and should therefore be extrapolated to human small-intestinal microbiology with caution. Further validation using complementary metabolic models and human studies is warranted. In addition, the present study focused primarily on microbiota- and SCFA-associated changes and did not directly examine microbiota-independent host responses to MPs. Immune-cell metabolism, particularly macrophage polarization and glutamine metabolism, is closely associated with obesity-related inflammation, insulin resistance, and T2DM [51]. Future studies should therefore investigate whether MPs influence macrophage metabolic programming and related inflammatory pathways. Potential host mechanisms, including hormetic activation of adaptive stress-response pathways, may also contribute to the observed metabolic improvements and warrant further investigation. The microbiota analysis focused on relative taxonomic composition. Furthermore, although the Greengenes 13_8 database has been widely used for 16S rRNA gene-based microbial annotation, it represents an earlier-generation reference database. Future studies incorporating updated reference databases may further improve taxonomic resolution and provide additional insights into microbial community characterization.

## 4. Conclusions

This study systematically mapped the spatial heterogeneity of cecal and colonic microbiota and temporal changes in fecal SCFA profiles in T2DM mice, providing insights into the multidimensional microecological changes associated with the anti-diabetic effects of MPs. Our results demonstrate that MPs ameliorated hyperglycemia and improved diabetes-associated metabolic abnormalities, accompanied by spatially heterogeneous alterations in cecal and colonic microbial communities. Specifically, within the sample cecal and colonic lumen compartments, MPs exhibited prebiotic-like microbial modulation characteristics, particularly in the ascending colonic lumen, where they were associated with increased relative abundance of beneficial genera such as *Allobaculum*, *Prevotella*, and *Oscillospira*. Concurrently, in the sampled cecal and colonic mucosal compartments, MPs were associated with reduced relative abundance of inflammation-associated taxa, including *Helicobacter*. This spatial pattern, characterized by alterations in luminal and mucosa-associated microbial communities, was accompanied by time-dependent changes in fecal SCFA profiles. Collectively, the improved metabolic phenotype following MP intervention was accompanied by spatial alterations in cecal and colonic microbial communities and time-dependent changes in fecal SCFA profiles. Our findings advance the current understanding of polyphenol–microbiota interactions from static, endpoint-based observations to a spatially resolved cecal and colonic microbial and temporally resolved fecal SCFA framework, and provide a basis for future investigation of the microbial and metabolic changes associated with MP intervention in T2DM.

## Data Availability

The original contributions presented in this study are included in the article. Further inquiries can be directed to the corresponding authors.
